# Cataract surgery under topical anesthesia: Gender-based study of pain experience

**DOI:** 10.4103/0974-620X.71893

**Published:** 2010

**Authors:** Sanjiv Kumar Gupta, Ajay Kumar, Swati Agarwal

**Affiliations:** Department of Ophthalmology, Chatrapati Sahuji Maharaaj University, Lucknow, India

**Keywords:** Cataract, gender, lignocaine jelly, manual small incision cataract surgery, pain, phacoemulsification, topical anesthesia

## Abstract

**Background:**

Pain perception, expression, tolerance, and the healthcare provider’s evaluation and management of pain are affected by the gender of the patient. To the best of our knowledge, there is lack of gender-based evaluation of pain during cataract surgery under topical anesthesia.

**Aims:**

This study has been initiated to evaluate and determine pain experience during cataract surgery under topical anesthesia and to study the gender-based differences of the same.

**Settings and Design:**

Hospital-based study involving cataract surgery under topical anesthesia using standard cataract surgery. It was an interventional comparative case series.

**Materials and Methods:**

One hundred and sixty patients were included in four groups, according to the gender and choice of surgery. Patients underwent either phacoemulsification with foldable intra ocular lens (IOL) or manual small incision cataract surgery with rigid IOL under topical anesthesia. Patients ranked their pain experience on VAS scale after the surgery and the surgeon recorded the ease/difficulty accordingly using a questionnaire.

**Statistical Analysis Used:**

MedCalc version 10.2.0.0 (www.medcalc.be) for windows was used to analyze the results. Analysis of variance (ANOVA) test and Kruskal-Walis test were used to analyze the data.

**Results:**

The overall visual analog scale (VAS) score was 0.8 (SD 1.3 range 0-8), with no statistical difference among the groups (*P*=0.5). The average surgeon’s score was 3.3 (SD 0.71 range 3-7), with no statistical difference between the groups (*P*=0.37).There were no sight threatening complications during the surgery in any group.

**Conclusions:**

The outcome of the study demonstrates that the patients undergoing cataract surgery under topical anesthesia perceive comparable pain and discomfort irrespective of their gender.

## Introduction

A common notion and belief is that there is gender-based difference for tolerance and response to pain. However, there are several contradictory reports in the literature. Some reports conclude that the pain tolerance and analgesic use is unaffected by gender,[1,2] others report that females report and perceive greater degree of pain when compared to male counterparts.[3–10] Health care provider’s evaluation of pain is also biased by the gender of the patient, but the reports in the literature are inconclusive due to contradictory reports. There are studies which report that physicians treat women less aggressively for their pain,[11] while others report that the female patients are perceived by providers to have more pain than male patients and female patients also receive more medications and stronger analgesics.[4]

The predictive model for pain experience is not simple and straightforward as pain sensitivity is thought to be mediated by socio cultural, psychological, and biological factors. Gender is an important variable and should be taken into account in both research and the clinical practice of pain management.[7] Thus evaluation of pain during any medical or surgical procedure has to be evaluated and analyzed specifically with no scope of a generalized formula applicable to all procedures.

Surprisingly, literature search done using ‘Scopus’ and ‘PubMed’ did not yield any result for gender- based pain evaluation among the patients undergoing cataract surgery under local or topical anesthesia, indicating the lack of research in this field. With this perspective, we initiated this study of evaluation of patients’ pain experience during cataract surgery under topical anesthesia with intracameral lignocaine supplementation using two different techniques (phacoemulsification and manual small incision cataract surgery). Topical anesthesia may be the choice owing to various advantages it offers in addition to safety for the target elderly population.[12] The aim of the study has been to evaluate the patients’ pain experience during cataract surgery under topical anesthesia with intracameral lignocaine, using two standard techniques and to do a gender-based analysis of the pain experience.

## Materials and Methods

A sample size of 40 patients per group was decided upon to detect a difference of 1 unit on average visual analog scale (VAS) scores among the groups (minimum sample size was calculated to be 37 patients per group keeping type I error rate 0.01; type II error rate 0.1; power = 0.9; standard deviation for VAS scores during cataract surgery was taken to be 0.97).[13] Approval by the institute ethical committee was taken before recruiting the patients in the study (ethics committee of Chatrapati Sahuji Maharaaj Medical University, Lucknow, U.P., India). The study was done at a single center and by a single surgeon.

Patients undergoing cataract surgery for visually disturbing uncomplicated cataract were included in the study. Patients were randomized for surgical technique (phacoemulsification or manual small incision cataract surgery) and then further segregated into groups according to their gender, thus forming four groups,

Group I: female gender undergoing phacoemulsification.
Group II: male gender undergoing phacoemulsification.
Group III: female gender undergoing manual small incision cataract surgery.
Group IV: male gender undergoing manual small incision cataract surgery.

Patients were consecutively recruited till there were 40 patients in each group who consented for topical anesthesia and the surgical technique offered. Exclusion criteria were lignocaine sensitivity, inability to understand and follow verbal commands, and any ocular disease other than cataract.

No blinding was employed as patients were aware of the surgical technique due to standard informed consent taken for surgery and the operating surgeon could not be blinded due to obvious reasons. Topical anesthesia was provided by using lignocaine jelly (Xylocaine 2% jelly Astra Zeneca, India) and supplemented by intracameral preservative free 0.5% lignocaine solution (prepared by four times dilution of xylocard 2% solution (Astra Zeneca, India) with ringer lactate solution). The safety and utility of the jelly formulation has been studied and demonstrated in several studies.[14] Jelly provides comparable[15,16] and possibly superior anesthesia,[17,18] wets the corneal surface, aids in pupillary dilatation without the use of sympathomimetics or parasympatholytic agents.[14] Adding intracameral lignocaine solution aids in increasing patient comfort and is safe and assists in pupillary dilatation.[19–22]

Manual small incision cataract surgery (MSICS) was done using sclero-corneal tunnel as described by Gupta *et al*, with 6.5 mm optic PMMA intra ocular lens (IOL).[13] Phacoemulsification was done with clear corneal 2.8 mm incision using direct chop technique with hydrophilic acrylic foldable lens implantation using cartridge and injector system. No sedation was given to the patients preoperatively.

**Figure 1:**
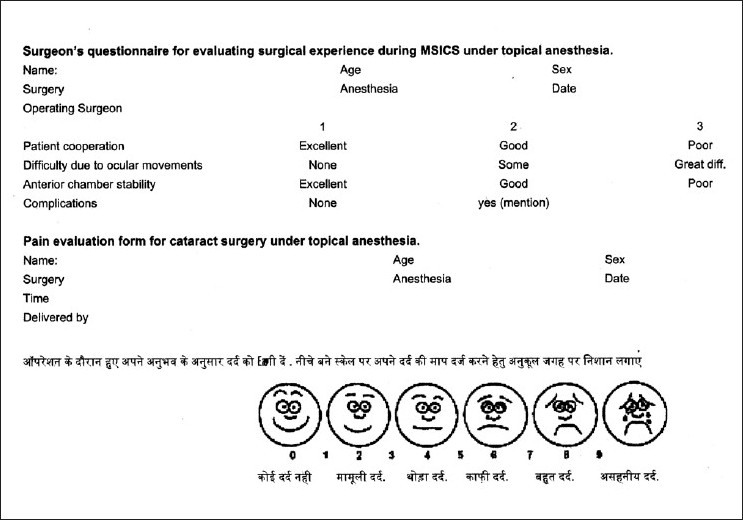
The pain evaluation form containing VAS and modified Wong scale to be filled by the patient after the surgery. The second part contains the surgeon’s questionnaire for evaluating his experience during the surgery of the same patient

During the surgery, the patients were instructed to communicate verbally regarding any pain during the surgery. This was recorded with the surgical step. At the end of the surgery, the eye was patched and patients were given a pain evaluation form containing modified Wong scale and a VAS for indicating the grade of pain experienced during the surgery [Figure 1]. Those patients, who could not read the form, were explained the questionnaire and verbal response was recorded by the ophthalmic assistant. The eye patch was removed after a period of 20 to 30 min and topical medications were initiated in all the groups.

The surgeon’s experience in terms of ease/difficulty was evaluated using a questionnaire presented to the surgeon just after the surgery. The questions were devised so that the patients’ cooperation, unwanted ocular movements and anterior chamber stability were evaluated on a three-point scale (one—three) with a lower grading indication more favorable experience by the surgeon. Thus the total score could range from three to nine. [Figure 1].

## Results

The study included 160 eyes of 160 patients, with patients divided into four groups as described earlier.

The average age of all the patients in the study was 60.08 years (SD 12.04, range 14-87 years). Descriptive analysis of the age distribution of subjects in each group is presented in Table 1. No statistical difference was demonstrated in the age distribution of subjects among the groups. (one way ANOVA (analysis of variance) test significance level *P*=0.58)

**Table 1:** Descriptive statistics of the age distribution of subjects in the four Groups

*All Subjects in Study*		*Group I*	*Group II*	*Group III*	*Group IV*
Mean (Age in years)	60.08125	61.2	58.9	58.625	61.6
Standard error	0.951867	1.365415	2.459883	1.813655	1.841056
Median	62	62.5	61	60	62
Mode	65	50	70	65	58
Standard deviation	12.04027	8.635645	15.55767	11.47056	11.64386
Sample variance	144.9682	74.57436	242.041	131.5737	135.5795
Range	73	38	71	48	66
Minimum	14	46	14	30	21
Maximum	87	84	85	78	87
Count	160	40	40	40	40
Confidence level (95.0%)	1.879934	2.761813	4.975583	3.668463	3.723887

Group I: Female patients undergoing phacoemulsification surgery. Group II: Male patients undergoing phacoemulsification. Group III: Female patients undergoing MSICS. Group IV: Male patients undergoing MSICS

**Figure 2:**
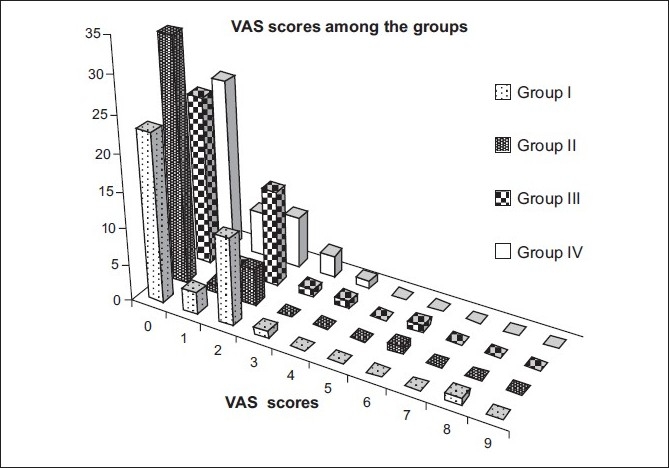
Bar chart demonstrating the VAS (visual analog scale) values for pain evaluation in the four groups. Group I: Female patients undergoing phacoemulsification surgery. Group II: Male patients undergoing phacoemulsification. Group III: Female patients undergoing MSICS. Group IV: Male patients undergoing MSICS

The overall VAS score was 0.8 (SD 1.3 range 0-8). The distribution of VAS score is illustrated in Figure 2. The descriptive statistics of the individual groups is depicted in Table 2. The average VAS score was lowest in the patient in group II (0.42 (male patients undergoing phacoemulsification)) and highest among the group III (1.0 (female patients undergoing manual small incision cataract surgery).

**Table 2:** Descriptive statistics of the VAS scores of subjects in the four groups

*All the patients in the study*		*Group I*	*Group II*	*Group III*	*Group IV*
Mean	0.8	0.95	0.425	1	0.825
Standard error	0.102944083	0.237373448	0.178625	0.217798	0.178625
Median	0	0	0	0	0
Mode	0	0	0	0	0
Standard deviation	1.302151099	1.501281504	1.129727	1.377474	1.129727
Range	8	8	6	6	4
Minimum	0	0	0	0	0
Maximum	8	8	6	6	4
Sum	128	38	17	40	33
Count	160	40	40	40	40

Group I: Female patients undergoing phacoemulsification surgery. Group II: Male patients undergoing phacoemulsification. Group III: Female patients undergoing MSICS. Group IV: Male patients undergoing MSICS

Painless surgery (VAS score zero) was reported by 57.5% (23 of 40) patients in the groups I, III, and group IV. However among group II patients 82.5% (33 of 40) reported painless surgery (VAS score zero). Those patients who experienced mild pain (VAS score ≤ 3)[23,24] in each group was as follows, group I, II, IV 97.5% (39 of 40) patients and 95% (38 of 40) patients in group III. Kruskal-Wallis test (nonparametric test to compare three or more unpaired groups) to test statistical difference between the VAS scores among the groups revealed no statistically significant difference among the groups (*P*=0.054).

**Figure 3:**
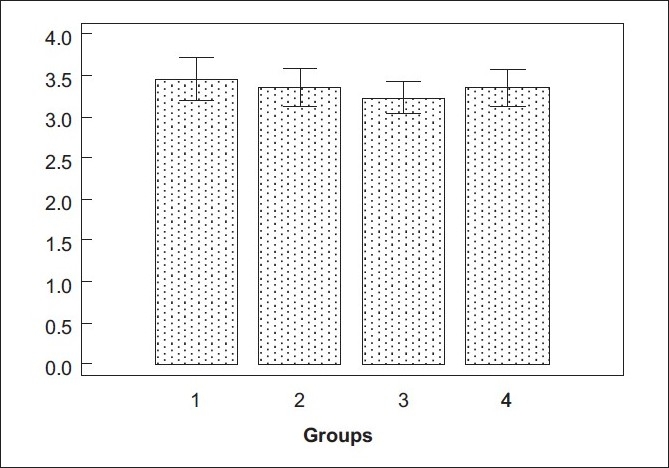
Box plot of the surgeon’s score in the four groups. Group I: Female patients undergoing phacoemulsification surgery. Group II: Male patients undergoing phacoemulsification. Group III: Female patients undergoing MSICS. Group IV: Male patients undergoing MSICS

The comparison graph of surgeon’s score for various groups is depicted in Figure 3. The average surgeon’s score was 3.3 on a scale of 3-9 (SD 0.71 Range 3-7). The descriptive analysis of the surgeon’s score in various groups is depicted in Table 3. The minimum average score (maximum ease during the surgery) was in group III (3.22) and maximum average score (maximum difficulty during the surgery) was in group II (3.45). D’Agostino-Pearson test for Normal distribution showed that the distribution was non-gaussian in all the groups (*P*<0.0001). Kruskal-Wallis test (non parametric test for comparing more than two groups) to detect the differences between the surgeon’s score among the groups showed that there was no statistical difference between the groups (*P*=0.37).

**Table 3:** Descriptive statistics of surgeon’s score of the subjects in the four groups

*All Patients*		*Group I*	*Group II*	*Group III*	*Group IV*
Mean	3.34375	3.45	3.35	3.225	3.35
Standard error	0.056806	0.128851	0.1163	0.097976	0.110651
Median	3	3	3	3	3
Mode	3	3	3	3	3
Standard deviation	0.718549	0.814925	0.735544	0.619657	0.699817
Range	4	4	3	3	3
Minimum	3	3	3	3	3
Maximum	7	7	6	6	6
Count	160	40	40	40	40
Confidence level(95.0%)	0.112192	0.260626	0.235239	0.198176	0.223812

Group I: Female patients undergoing phacoemulsification surgery. Group II: Male patients undergoing phacoemulsification. Group III: Female patients undergoing MSICS. Group IV: Male patients undergoing MSICS

The steps in MSICS when patients had discomfort, included intracameral injection of lignocaine solution, rotation and prolapse of nucleus from the capsular bag after hydrodissection, and irrigation aspiration of cortical matter and insertion of IOL. Patients undergoing phacoemulsification also felt discomfort during the same steps except for nucleus prolapse which was not done in the phacoemulsification group.

There was no sight threatening complication during the surgery in any group. Small descemets’ membrane detachment in vicinity of the internal opening of the incision was noted in six and five patients in group III and group IV (both MSICS groups) respectively. These were the patients in whom the nucleus was large and the nucleus was delivered in two or more pieces after getting fractured at the exit of the tunnel. Corneal burn of the anterior lip of the corneal incision was seen in seven patients in group I and 11 patients in group II, but self sealing of the incision and anterior chamber depth was maintained in all these patients and no suturing of the wound was needed. These patients had larger and harder nucleus and phacoemulsification time exceeded more than a minute resulting in wound burn. Sub-conjunctival hemorrhage was seen in majority of cases in manual small incision cataract surgery patients, at the conjunctival incision site and only in four patients undergoing phacoemulsification.

## Discussion

Gender-based evaluation of pain during management of medical conditions has been studied with contradictory reports.[1–10] Going by traditional and common understanding, female gender is associated with being fragile, more sensitive to pain and less tolerant to pain when compared to male gender. Being a common belief and understanding, the health care provider may also share the same view and there may be difference in the pain management provided.[4,11] During cataract surgery, pain management is not only important from patient’s point of view but also the surgical outcome may be adversely affected due to an uncooperative patient suffering from pain during the procedure.

The study has been aimed to detect gender-based differences between the pain experienced during cataract surgery done by two popular and standard techniques using topical and intracameral combined anesthesia. The results may aid in deciding the gender-based preference of surgical procedure under topical anesthesia. The secondary outcome has been to evaluate the safety of the techniques described.

The outcome of the study demonstrates that the patients undergoing cataract surgery under topical anesthesia perceive similar pain and discomfort irrespective of their gender. The female patients had more pain during the surgery in both the groups when compared to the male counterparts but this difference was not statistically significant.

Inter group comparison to evaluate the difference between the pain experienced by the patients undergoing phacoemulsification and manual small incision cataract surgery showed that though the average VAS was higher for manual small incision cataract surgery, there was no statistical difference between the groups.

The surgeon’s score with regard to the difficulty during the procedures was most favorable in group III (manual small incision cataract surgery, female gender). Interestingly this was the group with maximum VAS score. Thus analyzing further the group of patients with greatest discomfort were most cooperative during the surgery. This may sound contradictory but looking at the VAS scores we can conclude that these patients although had higher VAS scores but they still did not have intolerable pain and hence were relatively more alert and hence more cooperative, making it easier for the surgeon to operate upon. Overall the surgeon’s scores were statistically similar in all the groups. The described procedures were safe, with no sight threatening complications.

The study has been done at a single center and by a single surgeon; thus differences due to skill and personal bias cannot be excluded.

In conclusion, the study showed that both phacoemulsification and MSICS surgery can be done under topical anesthesia. The surgery is uncomplicated, safe and the pain during surgery is acceptable in majority of patients irrespective of the gender in both surgical groups. The surgeon’s score of difficulty during the surgery is also similar in all the groups with no statistical difference due to gender or the choice of surgical technique.
